# Supplementary material for: Meta-analysis of arterial anastomosis techniques in head and neck free tissue transfer
Source: PLoS One. 2021 Apr 1;16(4):e0249418. doi: 10.1371/journal.pone.0249418 (PMC8016284; doi:10.1371/journal.pone.0249418)
Supplement: S2 File — (DOCX) [file pone.0249418.s002.docx]

S2 File. The quality assessment results of the included studies.

| First author | Country | Publication Language | Newcastle-Ottawa Scale Score |
| --- | --- | --- | --- |
| Maisie L. Shindo | USA | English | 8 |
| Natalya Chernichenko | USA | English | 7 |
| 1. Wang | China | English | 8 |
| 1. Sun | China | Chinese | 7 |
| Z.Y. Yang | China | Chinese | 6 |
| 1. Guo | China | English | 7 |
